# Effect of gastric acid-suppressive therapy and biological variation of serum gastrin concentrations in dogs with chronic enteropathies

**DOI:** 10.1186/s12917-017-1233-y

**Published:** 2017-11-07

**Authors:** Romy M. Heilmann, Nora Berghoff, Niels Grützner, Nolie K. Parnell, Jan S. Suchodolski, Jörg M. Steiner

**Affiliations:** 1 College of Veterinary Medicine, University of Leipzig, An den Tierkliniken 23, 04103 Leipzig, DE Germany; 2 Gastrointestinal Laboratory, Texas A&M University, TAMU 4474, College Station, TX 77843-4474 USA; 3 Department of Pathobiology & Diagnostic Investigation, College of Veterinary Medicine, Michigan State University, 784 Wilson Rd, East Lansing, MI 48824 USA; 4 Farm Animal Clinic, Vetsuisse Faculty, University of Bern, Bremgartenstrasse 109a, CH, 3012 Bern, BE Switzerland

**Keywords:** Antihistamine, Biological variation, Canine, Hypergastrinemia, Proton pump inhibitor

## Abstract

**Background:**

Serum gastrin concentration can help diagnose gastrinomas in dogs if >3–10× the upper reference limit (URL), but antisecretory therapy and other conditions can also cause hypergastrinemia. Effects of antisecretory therapy (famotidine or ranitidine, omeprazole) on serum gastrin concentration in dogs with chronic enteropathy (CE) and its biological variation (BV) are unknown. Aim of the study was to evaluate serum gastrin in acid-suppressant-treated or -naïve CE dogs; test the association between serum gastrin and histopathologic findings in acid-suppressant-naïve CE dogs; and evaluate the BV of serum gastrin in dogs not receiving any gastric acid suppressive therapy. Samples from 231 dogs were used and serum gastrin was measured by chemiluminescence assay. Gastric and duodenal histologic lesions were evaluated and graded. BV of serum gastrin was evaluated in serial samples.

**Results:**

Serum gastrin concentrations were significantly higher in acid-suppressant-treated than acid-suppressant-naïve dogs (*P* = 0.0245), with significantly higher concentrations in proton pump inhibitor (PPI)- than H_2_-antihistamine-treated patients (*P* = 0.0053). More PPI- than H_2_-antihistamine-treated dogs had gastrin concentrations above URL (*P* = 0.0205), but not >3× nor >10× the URL. Serum gastrin concentrations correlated with the severity of gastric antral epithelial injury (*P* = 0.0069) but not with any other lesions or the presence/numbers of spiral bacteria in gastric biopsies. Intra- and inter-individual BV were 43.4 and 21.6%, respectively, in acid-suppressant-naïve dogs, with a reciprocal individuality index of 0.49 and a critical difference of ≥29.5 ng/L.

**Conclusions:**

Antisecretory (particularly PPI) treatment leads to hypergastrinemia in CE dogs, but the concentrations seen in this study are unlikely to compromise a diagnosis of gastrinoma. Use of a population-based URL for canine serum gastrin and a URL of ≤27.8 ng/L are appropriate.

## Background

Gastrin is a small peptide hormone that is produced by gastric and proximal duodenal G cells [1]. Gastrin acts on cells via the cholecystokinin-2 receptor stimulating gastric acid secretion and having trophic effects on the gastric mucosa [1, 2]. Measurement of serum gastrin has been suggested to be useful for the diagnosis of gastrinomas in dogs if increases in serum gastrin are in excess of three to ten times the upper limit of the reference interval (RI) [2–6].

Serum gastrin concentrations can also be affected by other conditions including hypercalcemia, chronic kidney disease, as gastrin clearance is primarily renal, administration of medications such as gastric acid-suppressants, feeding, and long distance racing^1^ [1, 3, 7–12]. Serum gastrin concentrations were also approximately twice as high in a small group of dogs with moderate to severe chronic lymphocytic-plasmacytic enteritis (LPE) compared to a group of dogs without gastrointestinal disease, and were higher in dogs with severe microscopic lesions in the stomach [13]. However, the effect of antisecretory therapy on hypergastrinemia was not evaluated in this study [13].

Effects of gastric *Helicobacter* spp. on gastrin expression and release in dogs have not been definitively elucidated. While no differences in plasma gastrin concentrations were seen between dogs colonized with *Helicobacter* spp. and specific-pathogen-free, *Helicobacter* spp.-free dogs [14], an enhanced basal gastrin release from canine antral G cells was detected after incubation with *Helicobacter pylori* extracts [15], and an increase in gastrin-mRNA expression in the fundus of the stomach has been demonstrated in dogs harboring virulent strains of *Helicobacter heilmannii* [16].

Investigations in healthy dogs revealed that serum gastrin concentrations transiently increase during either histamine type-2 receptor antagonist (H2A) or proton pump inhibitor (PPI) therapy^2^ [7, 10]. However, the effect of gastric acid-suppressant administration on serum gastrin concentration has not been reported in dogs with chronic enteropathies (CE).

Biological variability is an important measure in clinical pathology and consists of both intra- (within-subject) and inter-individual (between-subject) variation [17, 18]. While for most clinical chemistry analytes the components of biological variation derived from healthy subjects allows for inferences to be made in specific disease states [17, 18], for some biomarkers their interpretation can differ between health and disease [18]. To the authors’ knowledge, the biological variation of serum gastrin concentrations in dogs has been reported in neither health nor disease.

The aims of the study were (i) to compare serum gastrin concentrations between dogs with CE either administered an H2A and/or PPI and dogs with CE not receiving any gastric acid-suppressive treatment, and to determine whether the serum gastrin concentrations can distinguish dogs with CE from dogs with gastrinoma; (ii) to test the possibility of an association between serum gastrin concentrations and clinical disease severity or specific microscopic lesions in the stomach and duodenum; and (iii) to evaluate the biological variability of serum gastrin concentrations in dogs with CE. We hypothesized that (i) serum gastrin concentrations are higher in acid-suppressant-treated dogs with CE compared to those dogs not receiving any antisecretory treatment, and that serum gastrin can help to separate dogs with CE from dogs with gastrinoma (historical positive controls); (ii) serum gastrin concentrations are correlated with the severity of clinical signs and with microscopic lesions in the stomach and duodenum; and (iii) use of a population-based RI for serum gastrin concentration is appropriate.

## Methods

### Sampling population

**Fig. 1:**
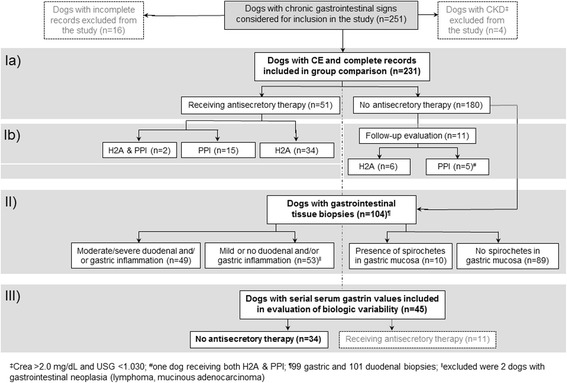
Flow chart that summarizes the subgroup allocation of all dogs (*n* = 231) included in the study. The three different parts of the study are indicated by the grey shaded areas: (I) evaluation of serum gastrin concentrations and antisecretory therapy in dogs with CE at the time of enrolment (Ia) and at serial evaluations (Ib); (II) evaluation of serum gastrin concentrations and histopathologic findings in dogs with CE; and (III) estimation of the biological variation of serum gastrin concentrations in dogs with CE

Dogs that presented for routine diagnostic evaluation of chronic (more than 2 weeks duration) gastrointestinal signs (i.e., vomiting, diarrhea, hypo- or anorexia, abdominal pain, weight loss), including a planned gastrointestinal endoscopy or exploratory laparotomy with collection of gastrointestinal tissue biopsies, and were diagnosed with CE [19] at the Veterinary Medical Teaching Hospitals at Texas A&M University (TAMU), Purdue University, or at one of several other referral hospitals across the United States, were prospectively enrolled between September 2009 and June 2015 (70 months) (see flow chart in Fig. 1).

The study was designed to evaluate several biomarkers, and was reviewed and approved by the Texas A&M University Institutional Animal Care and Use Committee (CRRC# 2009–06 and 2010–05, AUP# 2012-083A). The owner of each enrolled dog had to sign an informed consent form and was also asked to complete a study questionnaire, which included information about the dog’s clinical signs and patient history, including any current and past medications. The attending veterinarian was asked to assess the severity of the patient’s clinical signs by using a clinical disease activity scoring sheet with the nine criteria of the canine chronic enteropathy clinical activity index (CCECAI) scoring system [20].

Exclusion criteria were lack of complete patient records or clinicopathologic evidence of total hypercalcemia (>12 mg/dL) or renal azotemia (serum creatinine concentration > 2.0 mg/dL and urine specific gravity <1.030).

Follow-up data were included from patients that were clinically re-evaluated as described and complete sets of samples were collected at the time of recheck evaluation. Treatment of each individual dog, including the choice of diet, antibiotic, supplements, and/or anti-inflammatory or immunosuppressant therapy, was at the discretion of the attending clinician. Thus, gastric acid-suppressive treatment was not standardized. No interventions were performed as part of the study.

### Sample collection and analyses

Whole blood, serum, and urine samples were collected from all dogs at the time of presentation for diagnostic evaluation and, if reevaluated at the same veterinary hospital, at the recheck appointment or appointments. Whole blood and serum samples were collected after withholding food for a 12-h period, and were stored refrigerated until shipped to TAMU. Within 24 h of collection, all specimens, including gastrointestinal tissue biopsies (if obtained as part of the routine diagnostic work-up), collected outside of TAMU were sent to the TAMU Gastrointestinal Laboratory overnight on ice packs. Serum samples were processed as soon as received at the TAMU Gastrointestinal Laboratory, and were kept refrigerated in between analyses.

EDTA-anticoagulated whole blood was submitted for routine hematology (either at the institution recruiting the dog for the study or the Texas A&M Veterinary Medical Diagnostic Laboratory). Serum was used for a chemistry profile (using an automated clinical chemistry analyzer^3^) and analysis of several biomarkers. Urine samples were submitted for routine urinalysis (either at the institution recruiting the dog for the study or the TAMU Clinical Pathology service) and, if indicated, further diagnostic testing.

Serum gastrin concentration was measured following routine laboratory procedures (i.e., within 24 h after samples were received at the laboratory) using an automated commercially available chemiluminometric assay^4^^,^ 5 [21]. The lower limit of detection of the assay is 9.9 ng/L, and the intra- and inter-assay variability of the assay are <8% [21]. The upper reference limit (URL) of the assay has been previously established as ≤27.8 ng/L using serum samples from 41 healthy dogs^5^.

Histopathological analysis of gastrointestinal tissue biopsies was performed by one of 7 different board-certified pathologists through the TAMU Gastrointestinal Laboratory Histopathology service (median of 10 dogs evaluated by each pathologist, range: 1–28). Tissues were evaluated according to the World Small Animal Veterinary Association Gastrointestinal Standardization scoring system [19]. The severity of inflammatory and morphologic lesions in the stomach and duodenum was recorded using a 4-point grading system (0 = normal, 1 = mild lesions, 2 = moderate lesions, and 3 = severe lesions) with individual as well as composite scores being considered for analysis.

### Data analyses

All calculations and statistical analyses were performed using statistical software programs^6^^,^ 7 A Shapiro-Wilk *W* and Brown-Forsythe test were used to assess normality and equal variances, respectively. Summary statistics are presented as medians, interquartile ranges (IQR), and ranges. A Wilcoxon rank sum test (for unrelated groups) or Wilcoxon signed-rank test (for paired data) was used for two-group comparisons. A Spearman rank sum correlation coefficient (ρ) was calculated to assess potential relationships, with a Bonferroni correction for multiple comparisons applied if indicated. A likelihood ratio test served to evaluate the association between gastric acid-suppressive therapy and serum gastrin concentrations above RI. Significance was set a *P* < 0.05. Serum gastrin concentrations below the minimum detection limit of the assay (<9.9 ng/L) were assigned a concentration of 9.9 ng/L for all statistical analyses.

Receiver operating characteristic (ROC) curves were constructed to determine the sensitivities and specificities of serum gastrin concentrations to distinguish dogs with gastrinoma (historical positive controls [4, 22–31]) from acid-suppressant-naïve and/or acid-suppressant-treated CE dogs (disease controls). The Youden index was used to establish the optimum cut-off concentrations (expressed as fold elevation of the URL established for the respective assay that was used).

Intra- and inter-individual biological variation of gastrin concentrations in serum were assessed using serial samples from 45 dogs with CE (Fig. 1), with the analytical variation (CV_A_) set at 8% based on previous findings [21]. Following tests for outliers at 2 levels (i.e., intra- and inter-individual variation), a nested ANOVA model served to calculate the biologic coefficients of variation (CV): intra-individual (CV_I_), inter-individual (CV_G_), and total variation (CV_T_) [17]. The index of biological variation was calculated as reciprocal index of individuality (rII; rII = S_G_/S_A + I_), where a rII ≤0.7 suggests that a population-based RI is useful, whereas a rII ≥1.7 indicates that use of a population-based RI is not appropriate [17]. The minimum critical difference (MCD_0.05_) was determined as MCD_0.05_ = 2.77[dk_90_]^1/2^ (dk_90_ = 0.9 fractile of the observed distribution of within subject variance), which is the difference in concentration being significant at *P* < 0.05 [17].

## Results

### Study population

A total of 251 dogs were considered for inclusion into the study, of which 16 dogs had to be excluded due to incomplete records. An additional 4 dogs were excluded due to the presence of renal azotemia (Fig. 1), leaving a total of 231 dogs with CE (median age: 6.6 years; range: 0.3–17 years; 128 males/111 neutered and 103 females/90 spayed) included in the study. Breeds with *n* ≥ 5 dogs included: mixed breed dogs (*n* = 36), German Shepherd dogs (*n* = 20), Yorkshire terriers (*n* = 13), Labrador retrievers (*n* = 12), Boxers (*n* = 7), and Cavalier King Charles Spaniels, Golden retrievers, Jack Russell terriers, and Pitbull terriers (each *n* = 5).

Clinical signs at initial presentation were diarrhea in 171/231 dogs (74%), weight loss in 139/231 dogs (60%), vomiting in 128/231 dogs (55%), and hypo- or anorexia in 100/231 dogs (43%). The CCECAI at the time of enrolment into the study ranged from 1 to 19 (median: 7). Duration of clinical signs prior to enrollment in the study was 1–90 months (median: 5 months). At least one dietary trial had been performed in 161 dogs (70%) and an antibiotic trial in 95 dogs (41%), 46 dogs (20%) were given a probiotic and 23 dogs (10%) received cobalamin supplementation. Fourteen dogs were reported to have received corticosteroids prior to enrollment.

### Serum gastrin concentrations and gastric acid-suppressive therapy in dogs with CE

Serum gastrin concentrations were compared among 231 CE dogs with (H2A and/or PPI; *n* = 51) or without antisecretory therapy (*n* = 180). Serum gastrin concentrations at the time of study inclusion ranged from 9.9–571.0 ng/L (median: 10.3 ng/L, IQR: 9.9–19.6 ng/L), with an overall distribution of measurements skewed towards low serum gastrin concentrations. Information on serum gastrin concentrations and patient characteristics in this study has been summarized in Table 1. Serum gastrin levels were significantly higher in acid-suppressant-naïve dogs with severe or very severe clinical disease compared to those dogs with milder clinical signs (*P* = 0.0363).

**Table 1:** Summary of the serum gastrin concentrations and characteristics of the study population

Patient characteristics	Serum gastrin (in ng/L) in acid-suppressant-naïve dogs	*P*	Serum gastrin (in ng/L) in acid-suppressant-treated dogs	*P*
Age				
< 6 years	9.9 [9.9–14.9]	0.0770	10.6 [9.9–43.9]	0.5496
> 6 years	10.8 [9.9–22.8]		18.0 [9.9–34.4]	
Sex				
Female	9.9 [9.9–15.3]	0.5102	11.2 [9.9–31.8]	0.4276
Male	10.0 [9.9–19.4]		13.5 [9.9–44.6]	
Breed				
Purebred	10.1 [9.9–19.4]	0.1744	11.4 [9.9–35.0]	0.7206
Mixed breed	9.9 [9.9–14.4]		15.8 [9.9–46.9]	
German Shepherd (GSD)	12.1 [9.9–15.1]	0.8011	N/A	N/A
Non-GSD	9.9 [9.9–20.0]		11.4 [9.9–35.0]	
Yorkshire Terrier (YT)	11.3 [9.9–14.8]	0.6147	9.9 [9.9–9.9]	0.1202
Non-YT	10.1 [9.9–19.5]		13.5 [9.9–36.3]	
Clinical disease severity (CCECAI score)				
Mild to moderate	9.9 [9.9–14.8]	***0.0363***	11.2 [9.9–39.8]	0.9911
Severe to very severe	12.8 [9.9–25.0]		17.6 [9.9–31.2]	
Clinical signs				
Vomiting	9.9 [9.9–18.0]	0.6869	10.8 [9.9–33.1]	0.1354
No vomiting	9.9 [9.9–17.3]		22.8 [10.3–48.2]	
Diarrhea	10.0 [9.9–17.5]	0.5810	24.5 [9.9–12.4]	***0.0090***
No diarrhea	9.9 [9.9–16.3]		24.5 [9.9–50.0]	
Weight loss	9.9 [9.9–19.3]	0.6704	19.2 [9.9–44.7]	0.2113
No weight loss	10.3 [9.9–15.1]		10.7 [9.9–27.1]	
Hypo−/anorexia	10.4 [9.9–23.0]	0.5200	15.2 [9.9–31.8]	0.4983
No hypo−/anorexia	9.9 [9.9–16.1]		11.4 [9.9–47.5]	

*P* values in boldface and italicized indicate significance at *P* < 0.05

**Fig. 2:**
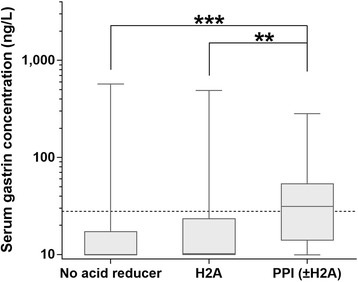
Serum gastrin concentrations and gastric acid-suppressive (PPI and/or H2A)-treatment in dogs with CE. Serum gastrin concentrations were significantly higher in dogs with CE administered either a PPI alone (*n* = 15) or in combination (*n* = 2) with an H2A (group PPI (±H2A); median: 31.2 ng/L, IQR: 14.0–54.1 ng/L, range: 9.9–282.0 ng/L) compared to CE dogs treated with an H2A alone (group H2A; n = 34; median: 10.2 ng/L, IQR: 9.9–23.7 ng/L, range: 9.9–490.0 ng/L; *P* = 0.0053) and those not receiving any gastric acid-suppressive treatment (group No acid reducer; *n* = 180; median: 9.9 ng/L, IQR: 9.9–17.4 ng/L, range: 9.9–571.0 ng/L; *P* = 0.0001). No significant difference was seen between H2A-treated CE dogs and dogs receiving no antisecretory treatment (*P* = 0.7312). Boxes: interquartile range (IQR), vertical lines within boxes: medians, whiskers: minimum to maximum data points; dashed line: upper limit of the RI (≤27.8 ng/L)

Serum gastrin concentrations were significantly higher in dogs with CE receiving an H2A and/or PPI (*n* = 51; median: 12.4 ng/L, IQR: 9.9–36.3 ng/L, range: 9.9–490.0 ng/L) compared to those not receiving antisecretory therapy (*n* = 180; median: 9.9 ng/L, IQR: 9.9–17.4 ng/L, range: 9.9–571.0 ng/L; *P* = 0.0245), with serum gastrin concentrations being significantly higher in CE dogs administered either a PPI (omeprazole) alone (*n* = 15) or in combination with an H2A (famotidine) (*n* = 2) compared to those dogs treated with an H2A alone (famotidine: *n* = 32, ranitidine: *n* = 2; *P* = 0.0053) or not receiving any antisecretory therapy (*P* = 0.0001) (Fig. 2). Further partitioning of the group of PPI-treated dogs was not possible given the small numbers of dogs. Gastric acid-suppressive therapy was significantly associated with serum gastrin concentrations above the URL (27.8 ng/L; 16/51, 31% vs. 28/180, 16%; *P* = 0.0151; for acid-suppressant-treated vs untreated dogs, respectively), but not above 3 × URL (83.4 ng/L; 4/51, 8% vs. 7/180, 4%, *P* = 0.2689) or above 10 × URL (278 ng/L; 2/51, 4% vs. 2/180, 1%, *P* = 0.2165), with a larger proportion of PPI-treated CE dogs compared to those given an H2A having a serum gastrin concentration above the URL (9/17, 53% vs. 7/34, 21%; *P* = 0.0205) but not above 3 × URL (3/17, 18% vs. 1/34, 3%, *P* = 0.0748) or above 10 × URL (1/17, 6% vs. 1/34, 3%; *P* = 0.6200). An effect of time (i.e., the study year) on serum gastrin concentrations was not detected in canine CE patients on antisecretory treatment (*P* = 0.3581) nor specifically in PPI-treated dogs (*P* = 0.1151).

The area under the ROC curve (AUROC), optimum cut-off levels, sensitivities, and specificities for serum gastrin concentrations to diagnose a gastrinoma are summarized in Table 2.

**Table 2:** Sensitivities and specificities at the optimal cut-off concentrations (expressed as the fold elevation above the respective URL for the assay) and area under the ROC curve (AUROC) for serum gastrin concentrations to distinguish acid-suppressant (H2A and/or PPI)-treated or acid-suppressant-naïve dogs with CE from dogs diagnosed with gastrinoma (*n* = 14 historical positive controls)

Group comparison	AUROC	Cut-off	Sensitivity	Specificity
all CE dogs vs. gastrinoma^a^	97%	>1.8 × URL	100%	90%
		>3.2 × URL	93%	95%
acid-suppressant-naïve CE dogs vs. gastrinoma^a^	98%	>1.8 × URL	100%	92%
		>3.2 × URL	93%	97%
acid-suppressant-treated CE dogs vs. gastrinoma^a^	95%	>1.8 × URL	100%	82%
		>10.3 × URL	57%	95%
H2A-treated CE dogs vs. gastrinoma^a^	97%	>1.8 × URL	100%	90%
		>3.2 × URL	93%	95%
PPI-treated CE dogs vs. gastrinoma^a^	91%	>3.2 × URL	93%	77%
		>10.3 × URL	57%	95%

*AUROC* area under the ROC curve, *CE* chronic enteropathy, *H2A* histamine type-2 receptor antagonist, *PPI* proton pump inhibitor, *URL* upper reference limit

^a^historical positive controls [4, 21–30]

Serum gastrin concentrations and the CCECAI scores in dogs not receiving an acid-suppressant (median: 5; IQR: 3–8, range: 1–19) were not correlated (ρ = 0.121, *P* = 0.1091). Also, no association was identified between serum gastrin concentrations and the presence of vomiting (*P* = 0.0675), diarrhea (*P* = 0.3646), weight loss (*P* = 0.1510), or hypo- or anorexia (*P* = 0.9416) in acid-suppressant-naïve dogs. Furthermore, serum gastrin concentration was not correlated with serum creatinine (median: 0.8 mg/dL; IQR: 0.6–1.0 mg/dL, range: 0.3–1.8 mg/dL; ρ = 0.0180, *P* = 0.8263) or serum total calcium concentrations (median: 9.6 mg/dL; IQR: 8.6–10.3 mg/dL, range: 3.4–13.0 mg/dL; ρ = −0.0437, *P* = 0.5928).

**Fig. 3:**
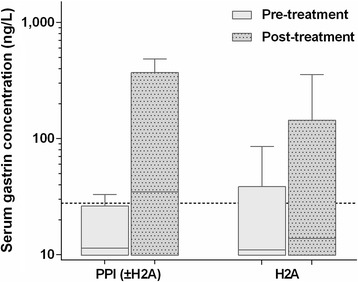
Paired serum gastrin concentrations were evaluated in initially acid-suppressant-naïve CE dogs that received either an H2A (*n* = 6) or a PPI alone or in combination with an H2A (*n* = 5) at their next recheck. Serum gastrin concentrations did numerically increase in those dogs receiving a PPI alone or in combination with an H2A (prior to antisecretory therapy: median = 11.4 ng/L, IQR = 9.9–19.6 ng/L, range = 9.9–33.1 ng/L; receiving gastric acid-suppressive therapy: media*n* = 34.8 ng/L, IQR = 9.9–254.0 ng/L, range = 9.9–485.0 ng/L), but the difference did not reach significance (*P* = 0.1563). No difference in serum gastrin concentrations was seen in dogs given an H2A alone (prior to antisecretory therapy: median = 11.0 ng/L, IQR = 9.9–20.2 ng/L, range = 9.9–85.5 ng/L; receiving acid-suppressant therapy: median = 13.9 ng/L, IQR = 9.9–59.6 ng/L, range: 9.9–355.0 ng/L; *P* = 0.5938). Boxes: interquartile range (IQR), vertical lines within boxes: medians, whiskers: minimum to maximum data points; dashed line: upper limit of the RI (≤27.8 ng/L)

Paired serum gastrin concentrations were evaluated in 11 initially acid-suppressant-naïve CE dogs that had received an H2A or a PPI alone or in combination with an H2A when rechecked 2–35 weeks (median: 13 weeks) after initiation of treatment. Dogs that were resampled had a median change (decrease) in CCECAI score of 83% (70% in H2A-treated dogs and 88% in dogs receiving a PPI with or without H2A). Serum gastrin concentrations did numerically increase in those dogs receiving a PPI alone or in combination with an H2A, but the difference did not reach significance (*P* = 0.1563) (Fig. 3); and no differences were seen in dogs given an H2A (*P* = 0.5938). There was no correlation between the change in CCECAI scores and the change in serum gastrin concentrations (ρ = −0.073, *P* = 0.8302), neither was an improvement in the CCECAI score associated with a normalization of serum gastrin concentration (*P* = 0.3852).

### Serum gastrin concentrations and histopathologic results in acid-suppressant-naïve dogs with CE

Gastrointestinal tissues were available for 104 dogs: endoscopic biopsies from 88 dogs (median number of gastric biopsies: 12, range: 2–34; median number of duodenal biopsies: 12, range: 2–29) and surgical biopsies from 16 dogs (median number of gastric biopsies: 2, range: 1–4; median number of duodenal biopsies: 1, range: 1–3). Two dogs were excluded from this part of the study due to the diagnosis of gastrointestinal lymphoma or a gastric mucinous adenocarcinoma.

No difference in serum gastrin concentrations were detected between acid-suppressant-naïve CE dogs with moderate or severe histologic lesions in the stomach (*n* = 22; median: 9.9 ng/L, IQR: 9.9–25.7 ng/L, range: 9.9–571.0 ng/L) and those dogs with none or mild gastric lesions (*n* = 75; median: 10.2 ng/L, IQR: 9.9–14.9 ng/L, range: 9.9–104.0 ng/L; *P* = 0.9815). Serum gastrin concentrations were slightly higher in dogs with moderate or severe microscopic lesions in the duodenum (*n* = 38; median: 12.1 ng/L, IQR: 9.9–32.7 ng/L, range: 9.9–111.0 ng/L) or stomach/duodenum combined (*n* = 49; median: 11.4 ng/L, IQR: 9.9–30.5 ng/L, range: 9.9–571.0 ng/L) compared to dogs with only mild or no such lesions (duodenum: *n* = 61, median = 9.9 ng/L, IQR = 9.9–14.3 ng/L, range = 9.9–571.0 ng/L; stomach/duodenum: *n* = 53; median = 9.9 ng/L, IQR = 9.9–13.5 ng/L, range = 9.9–72.3 ng/L) but neither difference reached statistical significance (*P* = 0.0543 and 0.0726, respectively). Serum gastrin concentration demonstrated a moderate positive correlation with the severity of surface epithelial injury in the gastric antrum (ρ = 0.324; unadjusted *P* = 0.0023, adjusted *P* = 0.0069), but was not correlated with any other morphologic or inflammatory microscopic findings (Table 3). Histopathologic evidence of atrophic gastritis was not reported in any of the dogs included in this study. A weak positive correlation with the composite duodenal lesion score did not reach statistical significance (ρ = 0.211; unadjusted *P* = 0.0362, adjusted *P* = 0.0724).

**Table 3:** Correlation of serum gastrin with variables of the histologic score for canine CE

Histologic findings	correlated with		serum gastrin concentration (ng/L)		
		N	Spearman *ρ*	unadjusted *P* ^†^	adjusted *P* ^#^
Stomach (composite score)		97	−0.1291	0.2075	0.4150
Fundus: Morphologic criteria (sum)		92	0.0885	0.4014	ns
- Surface epithelial injury		92	0.0517	0.6248	ns
- Gastric pit epithelial injury		92	0.0151	0.8866	ns
- Mucosal fibrosis		92	0.0167	0.8741	ns
Fundus: Inflammatory criteria (sum)		92	−0.0902	0.3923	ns
- Intraepithelial lymphocytes		92	−0.0434	0.6815	ns
- Lamina propria LPC		92	−0.1358	0.1966	ns
- Lamina propria eosinophils		92	0.0408	0.6993	ns
- Lamina propria neutrophils		92	−0.0938	0.3740	ns
- Lamina propria macrophages		92	−0.0938	0.3740	ns
- Lymphoid follicular hyperplasia		92	0.0079	0.9401	ns
Antrum: Morphologic criteria (sum)		86	0.0299	0.7844	ns
- Surface epithelial injury		86	0.3241	***0.0023***	***0.0069***
- Gastric pit epithelial injury		86	−0.1258	0.2483	ns
- Mucosal fibrosis		86	−0.0968	0.3754	ns
Antrum: Inflammatory criteria (sum)		86	−0.1489	0.1737	0.6948
- Intraepithelial lymphocytes		86	−0.0474	0.6650	ns
- Lamina propria LPC		86	−0.1969	0.0691	0.4146
- Lamina propria eosinophils		86	0.0140	0.8990	ns
- Lamina propria neutrophils		86	−0.1933	0.0745	0.4470
- Lamina propria macrophages		86	−0.0651	0.5512	ns
- Lymphoid follicular hyperplasia		86	−0.0815	0.4557	ns
Duodenum (composite score)		100	0.2108	***0.0362***	0.0724
Morphologic criteria (sum)		100	0.1178	0.2432	0.4864
- Villus stunting		100	0.1130	0.2629	ns
- Epithelial injury		100	0.0452	0.6554	ns
- Crypt distension		100	0.0966	0.3391	ns
- Lacteal dilation		100	0.1320	0.1906	0.9530
- Mucosal fibrosis		100	−0.0467	0.6444	ns
Inflammatory criteria (sum)		100	0.1239	0.2218	0.4436
- Intraepithelial lymphocytes		100	−0.0877	0.3855	ns
- Lamina propria LPC		100	0.0680	0.5013	ns
- Lamina propria eosinophils		100	0.1927	0.0560	0.2800
- Lamina propria neutrophils		100	0.1096	0.2779	ns
- Lamina propria macrophages		100	0.0663	0.5121	ns

Relationship between serum gastrin concentrations and the severity of morphologic and inflammatory histologic lesions of the stomach and duodenum in dogs with CE (*n* = 102)

*CE* chronic enteropathy, *LPC* lymphocytes/plasma cells, *N* sample size, *ns* non-significant with *P* = 1.0000; ^†^without correction (*P* < 0.05); ^#^after Holm-Bonferroni correction (*n* = 2, 4, or 5)

*P* values in boldface and italicized indicate significance at *P* < 0.05

Also, no significant difference in serum gastrin concentrations was seen between acid-suppressant-naïve CE dogs with spiral bacteria present in gastric biopsies (*n* = 10; median: 11.3 ng/L, IQR: 9.9–16.1 ng/L, range: 9.9–28.5 ng/L) and those dogs without (*n* = 89; median: 9.9 ng/L, IQR: 9.9–17.1 ng/L, range: 9.9–571.0 ng/L; *P* = 0.9154); nor was there a difference between dogs with large numbers of spiral bacteria (*n* = 5; median: 13.4 ng/L, IQR: 11.3–21.7 ng/L, range: 9.9–28.5 ng/L) and those with small numbers of spiral bacteria in gastric biopsies (n = 5; median: 9.9 ng/L, IQR: 9.9–14.8 ng/L, range: 9.9–19.6 ng/L; *P* = 0.1812). Presence and numbers of spiral bacteria in gastric biopsies were also not associated with the severity of microscopic lesions in the stomach (*P* = 0.5708 and 0.4870, respectively) nor in the duodenum (*P* = 0.6944 and 0.3406, respectively).

### Biological variation of serum gastrin concentrations in dogs with CE

**Fig. 4:**
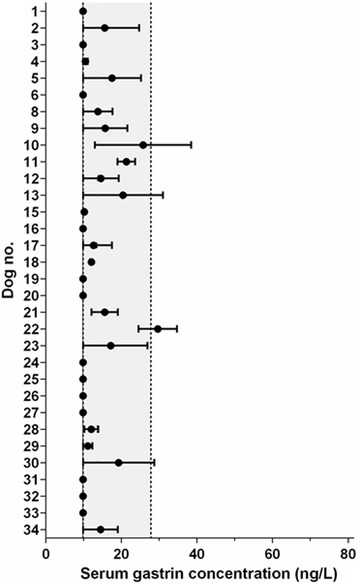
Biological variability of serum gastrin concentrations in dogs with CE that did not receive gastric acid-suppressive therapy. Serum gastrin concentrations ranged from 9.9–38.5 ng/L (mean: 13.5 ng/L) in 32 dogs with CE not receiving an acid-suppressant. A total of 44 measurements (60%) were at or below the detection limit of the gastrin assay and 4 (6%) were above URL. Closed circles: mean serum gastrin concentrations, horizontal bars: range of serum gastrin concentrations in individual dogs; gray shaded area (delineated by dashed vertical lines): RI (left dashed vertical line: lower reference limit coinciding with the lower detection limit of the assay

Biologic variability of serum gastrin concentrations was evaluated on an average of 2.3 serial specimens from 34 CE dogs (46 rechecks within 2–72 weeks, median: 7 weeks) not receiving any gastric acid-suppressive therapy (median age: 6.0 years; range: 0.3–12 years; 23 males/21 neutered and 11 females/9 spayed): 4 samples from 2 dogs, 3 samples from 8 dogs, and 2 samples each from 24 dogs. One within-subject outlier and two between-subject outliers were excluded from the dataset, resulting in a total of 73 mildly skewed data points and a narrow range of serum gastrin concentrations in individual dogs (Fig. 4a). CV_I_ was calculated as 43.4% and CV_G_ as 21.6%, resulting in an estimated CV_T_ of 73.0% and a rII of 0.49. Relevant serum gastrin concentration changes (as assessed by the MCD_0.05_) were calculated as ≥29.5 ng/L. The desirable analytical goal of estimated CV_A_ ≤ ½ × CV_I_ was achieved (maximum allowable imprecision ½ × CV_I_ = 21.7%).

Biologic variability could not be assessed in acid-suppressant-treated dogs (median age: 6.1 years; range: 2–12 years; 7 males/6 neutered and 4 females/4 spayed) due to the small number of data points (4 samples from 3 dogs, 3 samples from 2 dogs, and 2 samples each from 6 dogs) remaining for analysis after 2 between-subject outliers were excluded from the dataset.

## Discussion

This study evaluated serum gastrin concentrations in acid-suppressant-naïve CE dogs and CE dogs receiving gastric acid-suppressive therapy, and it is reasonable to assume that the findings of this study are likely to also translate to other species.

Antisecretory treatment with a PPI was associated with an approximately 3-fold increase (though not statistically significant) in serum gastrin concentrations (in contrast to an increase of only 1.3-fold in dogs receiving an H2A) in this population of dogs. This finding is consistent with the 3- to 5-fold increase seen in people taking a PPI [32]. The hypergastrinemia seen with H2A/PPI treatment can be explained by the reduction in gastric acidity, which inhibits the negative feedback on gastrin secretion [1].

Compared to Parente et al. [10], serum gastrin concentrations more than three-fold above the URL were rare in acid-suppressant-naïve (7/180, 4%) and H2A-treated CE dogs (1/34, 3%), but were seen in almost every fifth PPI-treated dog (3/17, 18%). This is consistent with PPIs being more potent at reducing gastric acid production by inhibiting all stimulatory effects through binding to the luminal α-subunit of the proton pump compared to H2A that block only the effect of histamine (but not gastrin and acetylcholine) as stimulators [1, 33]. Whether the length of PPI treatment or PPI dosing could also have an effect on serum gastrin concentration is unknown and cannot be clarified by the results of this study.

Serum gastrin concentrations in our study were comparable to those obtained in a small study in dogs with CE [13] but were slightly lower compared to serum gastrin concentrations measured in an experimental study in healthy dogs [10]. A possible explanation for the difference in measured concentrations is the use of different assays (a radioimmunoassay was used in the study by Parente et al. [10] whereas a chemiluminometric assay was used in the present study) as immunoassays depend on antigen-antibody binding and thus are not truly quantitative. An alternative explanation could be a difference in the length of antisecretory therapy as H2A administration has been shown to cause a transient hypergastrinemia in healthy dogs [10]. Our findings also agree with the hypergastrinemia seen in people with Crohn’s disease [34] whereas they are in contrast with another study that found systemic gastrin concentrations to be normal in Crohn’s disease patients [35]. The increase in serum gastrin concentrations in dogs with CE has been speculated to be due to either the gastric lesions per se or the stimulation of gastric G cells resulting from a decreased acidity in the gastric antrum [13]. However, the hypergastrinemia seen in CE dogs may also be associated with the inflammation as tumor-necrosis factor-α has been shown to affect gastrin concentrations [36, 37]. Whether decreased gastrin-releasing peptide receptor expression as seen in patients with Crohn’s disease [38] also affects the extent of the hypergastrinemia seen in dogs with CE has not been determined. While atrophic gastritis as a cause of hypergastrinemia [26] can be ruled out by histopathology (none of the dogs included in this study had histopathologic evidence of atrophic gastritis), the possibility of antral G-cell hyperplasia [33] (a condition that has not been reported in dogs to date) or an effect of antral distention associated with delayed gastric emptying or even gastric outlet obstruction on serum gastrin concentrations in individual dogs cannot be excluded in our study.

Clinical signs of dogs with gastrinoma can include vomiting, weight loss, abdominal pain, hypo- or anorexia, diarrhea, melena and/or hematochezia [3, 4, 22, 24, 26–31, 39, 40] and thus can mimic the clinical presentation of dogs with CE [13, 19, 20, 26]. Serum gastrin concentrations >3× or >10× above the URL (the currently used criteria for the diagnosis of gastrinoma) were rare in all dogs with CE (acid-suppressant-treated: 8% and 4%, respectively; untreated: 4% and 1%, respectively) in this study. Thus, the hypergastrinemia associated with CE and antisecretory therapy appears unlikely to compromise a diagnosis of gastrinoma in dogs, which differs from studies in people where a substantial overlap among those patient groups has been seen [41] and treatment with a PPI or H2A was associated with plasma gastrin concentrations being more than 20-fold higher than in acid-suppressant-naïve patients. Nonetheless, it is reasonable to assume that false positive results can also occur in dogs. Our findings also contrast an experimental study in dogs where supratherapeutic PPI doses caused up to 20-fold elevations of serum gastrin concentrations^2^.

Although the use of historical controls is controversial, with canine gastrinoma it was deemed necessary in order to determine the sensitivity and specificity of the gastrin assay due to the very low prevalence of this neoplastic condition [2–4, 29]. In the 14 canine gastrinoma cases reported in the literature to date where serum gastrin was measured [4, 22–31], the respective concentrations ranged from 72 to 2780 ng/L (median: 519 ng/L, IQR: 370–1020 ng/L; central 95th percentile: 90–2705 ng/L). Because the radioimmunoassays used in these 14 cases do not yield comparable results that could be used interchangeably with the chemiluminometric (Immulite®) method used in our study, the fold elevation of serum gastrin concentrations above the respective URL of the assay used in that study was evaluated and distinguished canine gastrinoma patients from both acid-suppressant-naïve and acid-suppressant-treated CE dogs with a high sensitivity and specificity. The best sensitivity was seen with cut-off serum gastrin elevations of 2- to 3-fold the URL, whereas a cut-off of 3- to 10-fold above the URL yielded the best specificity. However, the diagnostic performance of the serum gastrin assay in dogs with gastrinoma cannot be determined as the overall prevalence of the condition in dogs is unknown. Also, the impact of the use of PPIs on potentially delaying a diagnosis of gastrinoma or resulting in a false diagnosis of gastrinoma [42] in our canine population cannot be determined based on the results of this investigation. The optimal diagnostic algorithm to arrive at a diagnosis of gastrinoma [32, 43] in dogs will also require further study.

Consistent with our findings is the lack of a correlation between serum gastrin concentration and clinical disease activity seen in people with Crohn’s disease [34]. In contrast to a previous study in dogs that revealed significantly higher serum gastrin concentrations in dogs with severe gastric lesions compared to those dogs with no lesions in the stomach [13], serum gastrin concentrations correlated significantly only with the severity of surface epithelial injury in the gastric antrum but not with any other lesions in our study. A possible explanation for this discrepancy could be the different histologic lesion scores used or the selection of the patient population as only patients with moderate or severe gastric lesions were included in the study by García-Sancho et al. [13]. However, the possibility of an effect of the patient population, differences in assay methodology (e.g., radioimmunoassays are often more sensitive than chemiluminometric assays), or an effect of antisecretory drugs that was not considered in the study by García-Sancho et al. [13] cannot be excluded. Also, consistent with our results is the lack of a significant difference in serum gastrin concentrations between dogs with gastric carcinoma and healthy control dogs [44].

Only one dog with a large number of spiral bacteria had a serum gastrin concentration slightly above the URL (28.5 ng/L) but none of the dogs with spiral bacteria present had a serum gastrin concentration > 10× above URL in this study. Lack of an effect of spiral bacteria on serum gastrin concentrations and the presence of gastric or duodenal lesions is consistent with previous studies in dogs [14, 45] and also in cats [46]. This finding suggests that *Helicobacter*-like organisms in dogs may not inhibit the gastric proton pump (H^+^/K^+^-ATPase) as does *Helicobacter pylori* in people [1]. An interesting finding of our study is the normogastrinemia in one dog with gastric lymphoma (14.5 ng/L) and a dog with gastric mucinous adenocarcinoma (9.9 ng/L), which is in contrast to a previous case report [47]. Further, the prevalence of gastric spiral bacteria was lower in this study when compared to previous investigations [48, 49], but the prevalence of such organisms has been shown to vary with the patient population and method of detection [48]. An effect of prior antibiotic (given in 95 dogs included in this study) or probiotic administration (given to 46 of the dogs) on the prevalence of *Helicobacter*-like organisms [50–52] could also be possible.

Being a small peptide hormone, the serum half-life of gastrin is short (10–20 min) [1, 53] and the biological variation is important for the interpretation of serum gastrin concentrations. With the narrow range of serum gastrin concentrations in individual dogs with CE not receiving antisecretory therapy and the low rII, use of a population-based RI for serum gastrin concentrations appears justified. A relevant change in serum gastrin concentration of ≥29.5 ng/L indicates that the currently used URL in dogs (≤27.8 ng/L) is also appropriate.

This study had some limitations. First, while ultrasound evaluation (if performed as part of the diagnostic work-up) did not reveal any pancreatic masses (85% of dogs with a diagnosis of gastrinoma have pancreatic masses [3, 4, 25, 27, 30, 31] or evidence of metastatic disease [3, 29, 40]), a diagnosis of gastrinoma could not be definitively ruled out in all CE dogs. Also, gastric acid output was not determined as part of this study rendering the possibility of hypergastrinemia to exist in the face of either hypochlorhydria (appropriate hypergastrinemia) or hyperchlorhydria (gastric acid hypersecretion) unclear. Second, gastric acid-suppressive treatment was not standardized in dogs with CE enrolled in this study and the potency of gastric acid reducers (especially PPIs) has been shown to vary with the temporal relation to food intake [54] as well as the dose and dosing interval [1]. Thus, it is possible that most dogs were sampled at through. Furthermore, no constraints were placed on additional treatments in this study and a potential effect of supplements with reducing capacity (e.g., glutathione) on the potency of PPI [55] cannot be excluded. Third, involvement of different pathologists in the histopathological evaluation of tissue biopsies and of different attending veterinarians assessing the severity of the patients’ clinical signs in this study may potentially introduce an additional degree of variation. Not blinding the pathologist for the patient history, clinical, and/or clinicopathological findings is also a limitation. Fourth, serum samples could not be analyzed in one batch due to concerns about the stability of gastrin in these specimens over the 70-months study period. This practice has the potential to inflate the overall variation in the quantification of serum gastrin. Further, clear instructions on specimen handling procedures were given to participating institutions, but an effect of even slight differences in sample handling on serum gastrin concentrations cannot be entirely excluded. Lastly, a possible effect of undiagnosed mild hypercalcemia on serum gastrin concentrations cannot be entirely excluded as blood free (ionized) calcium was not measured in the dogs in this study. However, correcting the calcium concentration for serum albumin and total protein concentration, although of questionable value [56], did not change the results (data not shown) as none of the dogs were hypercalcemic after applying the correction formula.

## Conclusions

We conclude that, as seen in healthy dogs, antisecretory (particularly PPI) therapy leads to hypergastrinemia in dogs with CE, especially dogs with more severe duodenal and/or gastric lesions. However, the hypergastrinemia associated with gastric acid-suppressive therapy in CE dogs is unlikely to compromise a diagnosis of gastrinoma in dogs. Use of a population-based URL for serum gastrin and the currently used URL in dogs (≤27.8 ng/L) are appropriate.

## Abbreviations

AUROC: Area under the ROC curve
CCECAI: Canine chronic enteropathy clinical activity index
CE: Chronic enteropathy
CI: Confidence interval
CV: Coefficient of variation
CV_A_: Analytical variation
CV_G_: Inter-individual variation
CV_I_: Intra-individual variation
CV_T_: Total variation
H2A: Histamine type-2 receptor antagonist
IQR: Interquartile range
MCD_0.05_: Minimum critical difference
PPI: Proton pump inhibitor
RI: Reference interval
rII: Reciprocal index of individuality
ROC: Receiver operating characteristic
TAMU: Texas A&M University
URL: Upper reference limit

## Footnotes

1 Bregazzi VS, Fettman MJ, Twedt DC. Hypergastrinemia associated with hypercalcemia in the dog. J Vet Intern Med 2000; 14(3): 389 (abstract).

2 Dossin O, Jones K, Ridgway M, et al. Effect of omeprazole on serum gastrin concentrations and calcium metabolism in healthy dogs. J Vet Intern Med 2011; 25(3): 688 (abstract).

3 LiquiColor, Sirrus, Stanbio Laboratory, Boerne, TX, USA.

4 Immulite® 2000 Gastrin immunoassay, Siemens Healthcare Diagnostics Ltd., Deerfield, IL, USA.

5 Steiner JM, Findlay KM, Suchodolski JS. Analytical validation of a commercial immunoassay for the measurement of serum gastrin concentrations in dogs. J Vet Intern Med 2007; 21(6): 1438 (abstract).

6 JMP® v13.0, SAS Institute, Cary, NC, USA.

7 GraphPad Prism® v6.0, GraphPad Software, San Diego, CA, USA.
